# Molecular Identification and Phylogenetic Analysis of *Ascarids* in Wild Animals

**DOI:** 10.3389/fvets.2022.891672

**Published:** 2022-04-29

**Authors:** Shu-Yu Chen, Qi-Guan Qiu, Hai-Long Mo, Teng-Fang Gong, Fen Li, Jun-Lin He, Wen-Chao Li, Xin-Rui Xie, Wei Liu

**Affiliations:** ^1^Research Center for Parasites & Vectors, College of Veterinary Medicine, Hunan Agricultural University, Changsha, China; ^2^The Key Laboratory of Animal Vaccine & Protein Engineering, Changsha, China; ^3^Changsha Ecological Zoo, Changsha, China

**Keywords:** *Ascarids*, wild animals, mitochondrial DNA, ribosomal ITS, sequence analysis, phylogenetic relationship

## Abstract

Ascarid nematodes are the most common and harmful nematodes parasites in animals. By analyzing genetic variation, this study explores the genetic and phylogenetic relationship among ascarids from 11 different hosts. This study collected ascarid samples from the feces of nine animal species in Changsha Ecological Zoo of Hunan Province and two animal kinds in the College of Veterinary Medicine of Hunan Agricultural University. The mitochondrial gene (p*cox*1) and ribosomal ITS sequences were amplified, sequenced, and analyzed by PCR to identify the species of the samples. The phylogenetic tree was constructed based on two genes (*cox*1 and ITS) by the Neighbor-joining method, and the phylogenetic relationship was analyzed. The sequencing results showed that the sequence lengths of p*cox*1 and ITS genes in the samples were 441 bp and 838–1,177 bp, respectively. The difference rates were 0.00–1.70% in p*cox*1 gene and 0.00–7.30% in ITS gene. Phylogenetic analysis showed that ascarid worms from the white lion, Northeast tiger, South China tiger and cheetah were identified as *Toxascaris leonina*. Ascarids from the zebra were identified as *Parascaris equorum*, while those from chicken and peacocks were identified as *Ascaridia galli*. Ascarids of wolf and dog origin were *Toxocara canis*, the snake ascarids belonged to *Ophidascaris filaria*, and the bear ascarids belonged to *Baylisascaris transfuga*. There was a significant gap between different kinds of ascarid worms. We found that these two mitochondrial genes p*cox*1 and ITS showed a common characteristic that the intraspecific differences were significantly smaller than the interspecific differences, confirming that these two genes could be used as interspecific genetic markers for molecular identification of different ascarids origins. The intraspecific variation rate of the ITS gene was higher than that of p*cox*1, indicating that ITS can also be used in the genetic research of Ascaris species development. This study revealed the genetic evolution and phylogeny of ascarids in wild animals, and our results will help prevent and control ascarids in wild animals.

## Introduction

*Ascarid* nematodes are the most common and harmful internal parasites in animals, infecting all kinds of animals. Due to environmental factors and repeated infection, there are many kinds of ascarids in animals, and the infection intensity is high (1). *Ascarids* nematodes are mainly parasitic in the intestinal tract of animals and can cause symptoms such as weight loss, anemia, anorexia, diarrhea, abdominal pain, or intestinal dysfunction. In severe cases, this roundworm can cause intestinal obstruction or intestinal rupture. Ectopic parasitism can cause liver necrosis, pancreatic hemorrhagic necrosis, and pneumonia, which seriously threaten the host's life. In addition, following ingestion of infectious eggs, larvae can cause tissue damage and inflammatory reaction when they migrate into human and animals tissue. Ascarid larvae of animal origin can also migrate to the eyes and brain tissues of abnormal hosts (human beings), leading to visceral larval migration, ocular larval migration, and neurolarval migration, leading to neurological symptoms, visual impairment, or blindness. Moreover, parasites with neuroinvasive stages, such as *Toxocara canis*, can cause detrimental damage to the brain of intermediate or paratenic hosts (2). Therefore, ascarid nematodes possess a paramount zoonotic concern (3, 4). Identifying different ascarids is conducive to preventing and treating *Ascarids* infection in wild animals.

Due to the genetic variation caused by host and geographical environment, the limitations of ascarid morphological classification and identification are becoming more and more apparent. Mitochondrial DNA is an extracellular circular genetic material that, compared with nuclear genes, has a small molecular weight, simple structure, fast evolution rate, and lack of recombination (5). The mitochondrial gene cytochrome C oxidase subunit 1 (*cox*1) is relatively conservative, suitable for parasite classification, identification, and phylogenetic analysis (6). ITS is an internal transcribed spacer mediated by ribosomal DNA (rDNA) between 18S and 28S, including ITS-1 and ITS-2 sequences. This sequence has a fast evolution speed and small length. Coupled with coevolution, this fragment is consistent among different genome units, suitable for various molecular operations (7). Using genetic markers in rDNA can accurately solve taxonomic problems (8). Blouin (9) believes that using *cox*1 and *nad*4 genes in mtDNA is more effective as genetic markers to identify ascarids species, especially hidden species. Zhu et al. (10) amplified and sequenced the rDNA internal transcribed spacer (ITS) of *Toxocara canis, Toxocara felis*, and a kind of *Toxocara lumbricoides* from Malaysia by PCR. These ascarids could be classified by using ITS as a genetic marker (10). Presently, some researchers have used mitochondrial genes (*cox*1, *cyt*b, and *nad*1) and ribosomal genes (ITS and 5.8S) for species identification and intraspecific genetic variation of *Ascarids* (11–15). To explore the genetic evolution relationship between ascarids from nine wild animal species in Changsha Ecological Zoo, Hunan Province and ascarids from chicken and dogs in the College of Veterinary Medicine, Hunan Agricultural University. The ascarid worm species were determined by amplifying the p*cox*1 and ITS genes, and their genetic evolution relationship with other ascarids was analyzed. This study would further support wild animals'molecular epidemiology and population genetics of ascarid worms.

## Materials and Methods

### Sample Collection

The *Ascarid* samples in this study were mainly collected from animals of the Changsha Ecological Zoo in Hunan Province, including white lions (No.: WL1, WL2, Wl3), Northeast tigers (No.: ST1, ST2, ST3), South China tigers (No.: SCT1, SCT2, SCT3), cheetahs (No.: AJ1, AJ2, AJ3), zebras (No.: PE1, PE2, PE3), peacocks (No.: P1, P2, P3), wolfs (No.: CL1, CL2, CL3), snakes (No.: S1, S2, S3), and bears (No.: B1, B2, B3). Chicken (No.: A1, A2, A3) and dogs (No.: C1, C2, C3) parasite samples were collected from the College of Veterinary Medicine of Hunan Agricultural University. After washing, the worms were stored in 70% alcohol at −20°C.

### DNA Extraction and Enzymatic Amplification

A two cm part of each sample was cut and repeatedly washed with double distilled water six times for 5 min each time. Sterile tweezers were used to put the moistened sample into a 1.5 mL Eppendorf centrifuge tube, and 200 μL GA buffer solution and 20 μL protease K solution were added and mixed and incubated in a 37°C constant temperature water bath for overnight digestion. The digested worm suspension was used to extract DNA according to Tiangen genomic DNA extraction kit (Beijing, China). The PCR amplification system was 25 μL: 12.5 μL Taq PCR Master Mix (Bao Bioengineering Co., Dalian, China), 9.5 μL ddH_2_O, upstream and downstream primers (100 pmol /μL, 0.5 μL each), and 2 μL template DNA. At the same time, double distilled water was used as blank control instead of template DNA. Two pairs of primers designed in previous studies (16, 17) were used to amplify p*cox*1 and ITS genes. PCR amplification conditions were as follows: pre denaturation at 94°C for 5 min; denaturation at 94°C for 30 s, annealing at 50°C for 30 s, and extension at 72°C for 30 s, 35 cycles; and final extension at 72°C for 5 min. After recovery and purification by TaKaRa MiniBEST Agarose Gel DNA Extraction Kit Ver.4.0, the PCR products were sequenced by Beijing Qingke Xinye Biotechnology Co., Ltd.

### Sequencing and Phylogenetic Analysis

The sequencing results were analyzed by DNAstar 5.0 software, and the p*cox*1 and ITS sequences of ascarids from different hosts were compared with those of other *Ascaris* in GenBank. We used DnaSP V6.12 software to calculate the sequence haplotypes, nucleotide diversity, haplotypes diversity, and the average number of nucleotide differences. Using Mega 7.0 software, Kimura 2-parameter model, NJ method (Neighbor-Joining) was used to draw the phylogenetic tree, and the bootstrap test was calculated based on 1,000 bootstrap replicates.

## Results

### PCR Amplification

The sequence lengths of p*cox*1 and ITS genes of ascarids from different hosts were about 450 bp and 1,000 bp, respectively, which was consistent with the expected fragment length (Supplementary Figures 1, 2), and there was no nonspecific band.

### Genetic Characterizations of Ascarids

After comparison and correction, 33 different homologous ascarid samples in this study belonged to *T. leonina, P. equorum, A. galli, T. canis, O. filaria*, and *B. transfuga*. The amplification results of p*cox*1 and ITS genes are shown in Table 1. The sequences of p*cox*1 and ITS genes obtained by sequencing have been uploaded to the GenBank database, and the accession number of p*cox*1 sequences are OM867278-OM867307 and OM901120-OM901122. The accession numbers of ITS sequences are OM876348-OM876380.

**Table 1:** The amplification length results of p*cox*1 and ITS genes.

**S**	**Host**	**Species**	**nt size (bp)**	**A + T (%)**
p*cox*1	White lion Northeast tiger South China tiger Cheetah	*Toxascaris leonina*	441	66.9–67.1
	Zebra	*Parascaris equorum*	441	61.6–62.2
	Peacock Chicken	*Ascaridia galli*	441	66.4–66.6
	Wolf Dog	*Toxocara canis*	441	63.3–64.2
	Snake	*Ophidascaris filaria*	441	64.3–64.8
	Bear	*Baylisascaris transfuga*	441	64.2–64.6
ITS	White lion Northeast tiger South China tiger Cheetah	*Toxascaris leonina*	861–864	58.1–58.3
	Zebra	*Parascaris equorum*	838–840	61.9–62.0
	Peacock Chicken	*Ascaridia galli*	984–989	63.6–63.7
	Wolf Dog	*Toxocara canis*	1,159–1,177	50.4–51.0
	Snake	*Ophidascaris filaria*	1,038–1,039	53.3–53.4
	Bear	*Baylisascaris transfuga*	867–869	58.3–58.4

*S, number of polymorphic sites*.

Further nucleotide sequence homology analysis is shown in Table 2. The main differences were base exchange and base deletion.

**Table 2:** The nucleotide sequence homology analysis of p*cox*1 and ITS genes.

**S**	**N**	**n**	**Species**	**Different rate (%)**	**Intraspecific variation (%)**	**Homology (%)**
p*cox*1	WL1 WL2 WL3 ST1 ST2 ST3 SCT1 SCT2 SCT3 AJ1 AJ2 AJ3	12	*Toxascaris leonina*	0.0–2.3	0.0–5.4	87.8–100.0
	PE1 PE2 PE3	3	*Parascaris equorum*	0.0–1.7	0.07–3.2	84.1–100.0
	P1 P2 P3 A1 A3	5	*Ascaridia galli*	0.0–0.02	0.0–0.2	87.1–100.0
	CL1 CL2 CL3 C1 C2 C3	6	*Toxocara canis*	0.0–2.7	0.0–2.7	87.3–100.0
	S1 S2 S3	3	*Ophidascaris filaria*	0.0–0.9	0.0–0.5	87.3–100.0
	B1 B2 B3	3	*Baylisascaris transfuga*	0.0–1.4	0.0–6.0	86.0–100.0
ITS	WL1 WL2 WL3 ST1 ST2 ST3 SCT1 SCT2 SCT3 AJ1 AJ2 AJ3	12	*Toxascaris leonina*	0.0–0.1	0.0–0.1	68.7–100.0
	PE1 PE2 PE3	3	*Parascaris equorum*	0.0–0.1	0.0–0.7	50.1–100.0
	P1 P2 P3 A1 A2 A3	6	*Ascaridia galli*	0.0–0.7	0.0–0.8	52.2–100.0
	CL1 CL2 CL3 C1 C2 C3	6	*Toxocara canis*	0.0–7.3	0.0–7.3	52.3–99.8
	S1 S2 S3	3	*Ophidascaris filaria*	0.0–0.3	0.0–0.3	55.0–100.0
	B1 B2 B3	3	*Baylisascaris transfuga*	0.0–0.1	0.0–6.3	52.8–100.0

*S, number of polymorphic sites; N, number of isolates; n, sample numbers*.

### Phylogenetic Analysis

**Figure 1:**
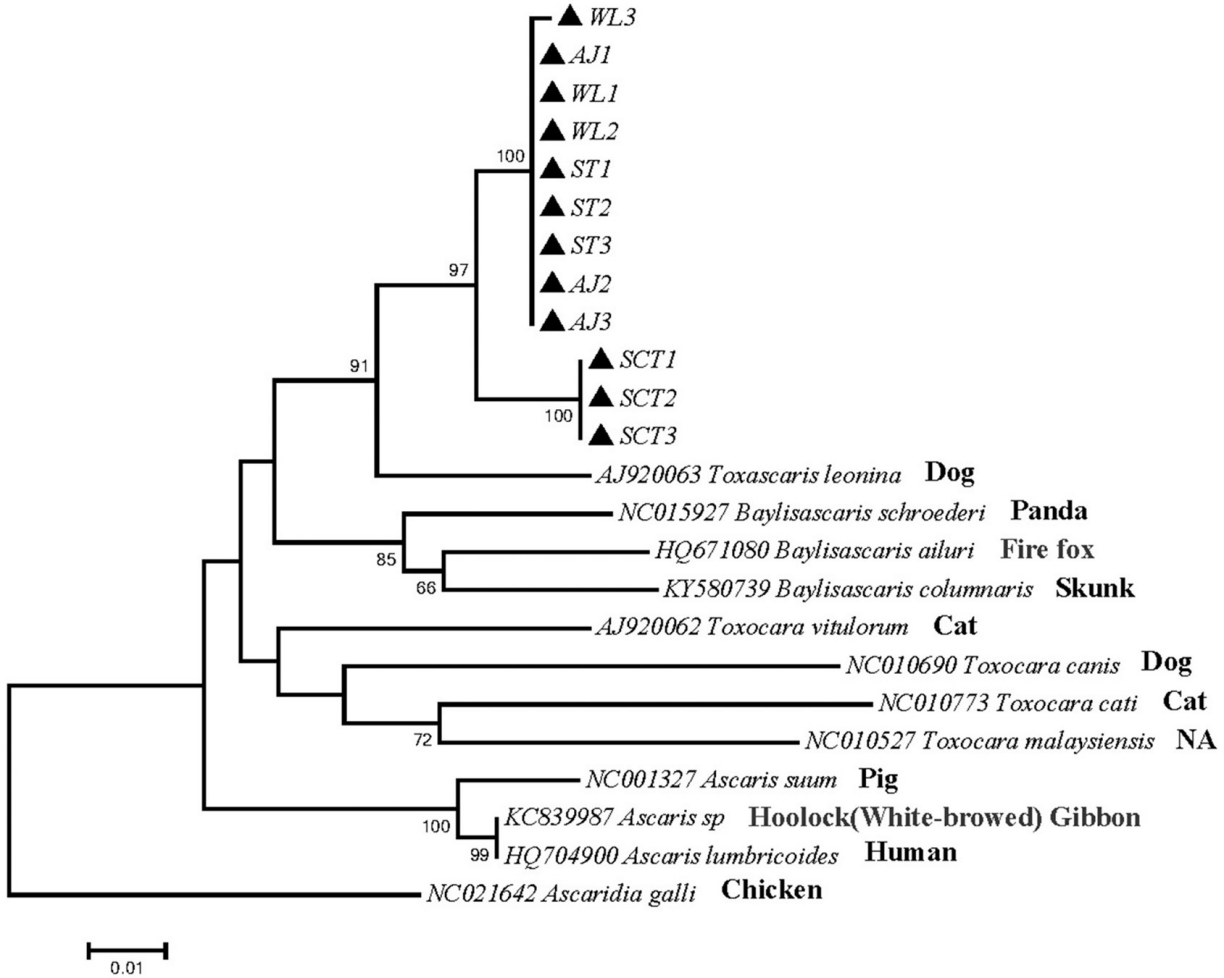
Neighbor-Joining estimatesof the phylogenetic relationships of ascarids based on p*cox*1 gene computed in MEGA version 7.0 under the Kimura 2-parameter model; The confidence levels in each node were assessed with the bootstrap method (1,000 pseudoreplicates) and bootstrap values >50; The genotypes identified in this study are indicated by filled triangle.

Phylogenetic tree 1 was constructed by using the p*cox*1 gene to explore the genetic distance of the p*cox*1 gene of ascarid worm from different host sources within and between species (Figure 1), which was in the same branch as *T. leonina* (NC023504) but far away from the branches of other ascarids, such as *Baylisascaris schroederi* (NC015927), *Baylisascaris ailuri* (HQ671080), *Baylisascaris columnaris* (KY580739), *Toxocara vitulorum* (AJ920062), *T. canis* (NC010690), *Toxocara cati* (NC010773), *Toxocara malaysiensis* (NC010527), *Ascaris suum* (NC001327), and *Ascaris lumbricoides* (HQ704900).

**Figure 2:**
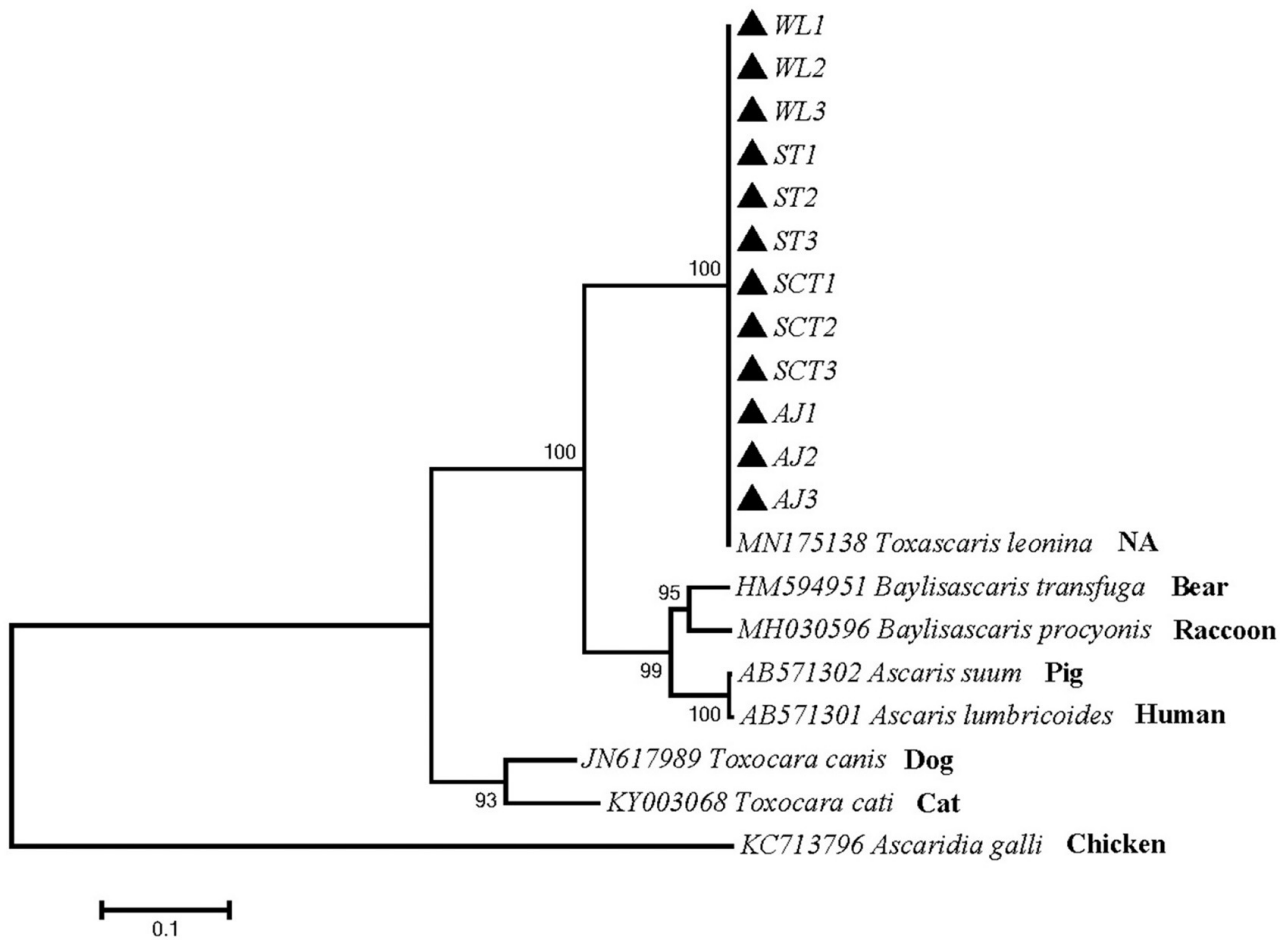
Neighbor-Joining estimates of the phylogenetic relationships of ascarids based on ITS gene computed in MEGA version 7.0 under the Kimura 2-parameter model; The confidence levels in each node were assessed with the bootstrap method (1,000 pseudoreplicates) and bootstrap values >50; The genotypes identified in this study are indicated by filled triangle.

Phylogenetic tree 2 was constructed by using the ITS gene to explore the genetic distance of the ITS gene of ascarid worm from different host sources within and between species (Figure 2), which was in the same branch as *T. leonina* (MN175138) and closely related to *B. transfuga* (HM594951), *Baylisascaris procyonis* (MH030596), *A. suum* (AB571302), and *A. lumbricoides* (AB571301). The difference between this sequence and *T. canis* (JN617989) and *T. cati* (KY0030684) was noticeable.

**Figure 3:**
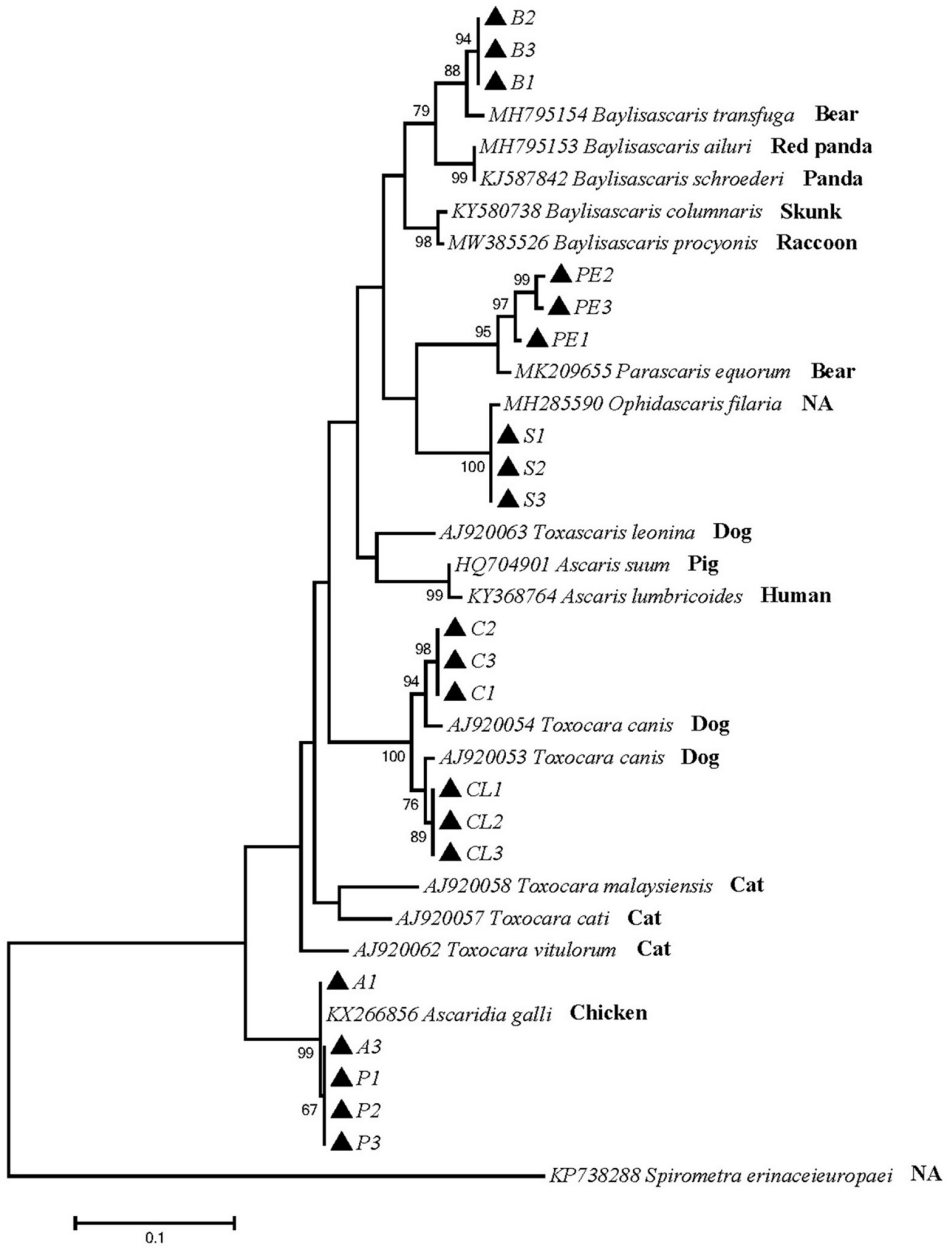
Neighbor-Joining estimates of the phylogenetic relationships of ascarids based on p*cox*1 gene computed in MEGA version 7.0 under the Kimura 2-parameter model; The confidence levels in each node were assessed with the bootstrap method (1,000 pseudoreplicates) and bootstrap values >50; The genotypes identified in this study are indicated by filled triangle.

Phylogenetic tree 3 was constructed using the p*cox*1 gene to explore the genetic distance of the p*cox*1 gene of ascarid worm from different host sources within and between species (Figure 3), and sequences PE1, PE2 and PE3 were in the same branch as *P. equorum* (MK209655). Sequences A1, A3, P1, P2, and P3 were in the same branch as *A. galli* (KX266856) and were closely related to *T. vitulorum* (AJ920062). Sequences C1, C2, C3, CL1, CL2 and CL3 were in the same branch with *T. canis* (AJ920054) and *T. canis* (AJ920053). Sequences S1, S2 and S3 were in the same branch as *O. filaria* (MH285590). Sequences B1, B2 and B3 were in the same branch as *B. transfuga* (MH795154), and closely related to other ascarids, such as *B. ailuri* (MH795153), *B. schroederi* (KJ587842), *B. columnaris* (KY580738), and *B. procyonis* (MW385526). Therefore, *B. transfuga, P. equorum* and *O. filaria* were closely related to *T. leonina* (AJ920063), *A. suum* (HQ704901), and *A. lumbricoides* (KY368764). These sequences were significantly different from other ascarids, such as *T. cati* (KY003068) and *T. malaysiensis* (AJ920058).

**Figure 4:**
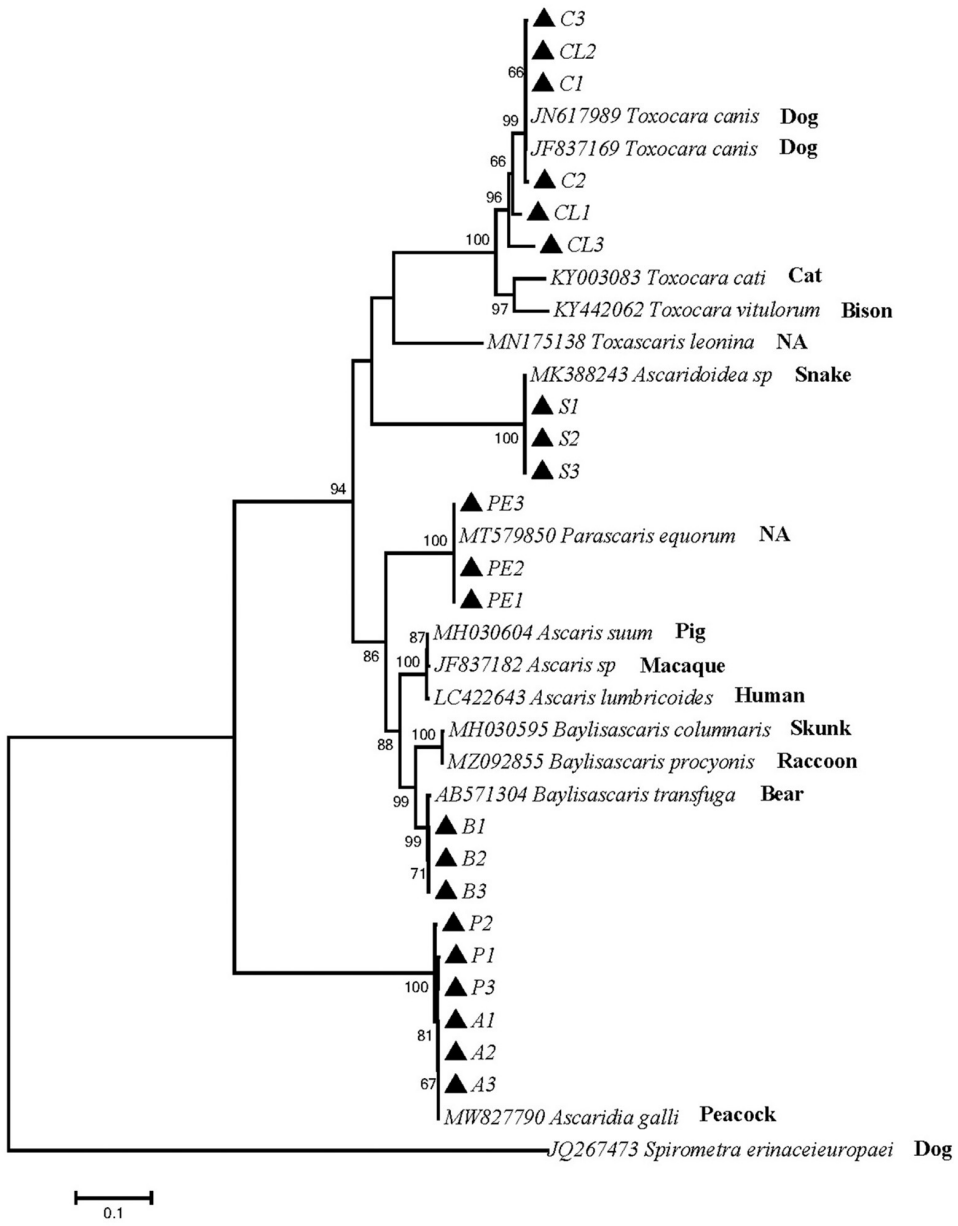
Neighbor-Joining estimates of the phylogenetic relationships of ascarids based on ITS gene computed in MEGA version 7.0 under the Kimura 2-parameter model; The confidence levels in each node were assessed with the bootstrap method (1,000 pseudoreplicates) and bootstrap values >50;The genotypes identified in this study are indicated by filled triangle.

Phylogenetic tree 4 was constructed by using the ITS gene to explore the genetic distance of the ITS gene of ascarid worms from different host sources within and between species (Figure 4), and sequences PE1, PE2 and PE3 were in the same branch as *P. equorum* (MT579850). Sequences A1, A2, A3, P1, P2 and P3 were in the same branch as *A. galli* (MW827790). Sequences C1, C2, C3, CL1, CL2 and CL3 were in the same branch with *T. canis* (JN617989 and JF837169) and closely related to *T. cati* (KY003083), *T. vitulorum* (KY442062), and *T. leonina* (MN175138). Sequences S1, S2 and S3 were in the same branch as *O. baylisi* (MW837142). Sequences B1, B2 and B3 were in the same branch as *B. transfuga* (AB571304) and closely related to *B. columnaris* (MH030595) and *B. procyonis* (MZ092855). In addition, *T. canis, T. cati, T. vitulorum*, and *O. baylisi* were closely related to *T. leonina* (MN175138). *B. transfuga, P. equorum, A. suum* (MH030604), *A. lumbricoides* (LC422643) were closely related. These sequences were significantly different from other ascarids.

## Discussion

In this study, ascarid samples from different 11 hosts were compared and analyzed for the first time. The amplified 33 different homologous ascarid samples belonged to *T. leonina, P. equorum, A. galli, T. canis, O. filaria*, and *B. transfuga*. And the two mitochondrial genes p*cox*1 and ITS showed the commonness that the intraspecific difference is significantly less than the interspecific difference. These results were consistent with those of Jin (18), He et al. (19), He et al. (11), Xie et al. (20), Huang et al. (21), and Sun (22). Our results confirm that the interspecific variation of the p*cox*1 and ITS genes in ascarids samples from different hosts was more significant than the intraspecific variation.

Nucleotide diversity is an essential indicator of mitochondrial genetic variation in a population. Nucleotide diversity considers the proportion of various mitochondrial haplotypes in the population. Thus, it is more accurate than the simple average genetic distance in reflecting the degree of mitochondrial polymorphism within a population. For most animals, PI values (nucleotide diversity) above 0.01 are considered to be highly variable (23). According to the analysis in Table 3, the two mitochondrial genes p*cox*1 and ITS showed the commonness that the intraspecific difference is significantly less than the interspecific difference, indicating that these two genes can be used as interspecific genetic markers for the molecular identification of different homologous ascarids. At the same time, the intraspecific variation rate of the ITS gene was higher than that of p*cox*1, suggesting that it can also be used for genetic research, such as ascarid germline development and molecular identification.

**Table 3:** Sequence analysis of nucleotide polymorphisms by DnaSP software.

**S**	**Species**	**N**	**H**	**Hd**	**π**	**K**
p*cox1*	*Toxascaris leonina*	12	3	0.53 ± 0.136	0.00873 ± 0.00269	3.848
	*Parascaris equorum*	3	3	1.000 ± 0.272	0.01395 ± 0.00473	6.333
	*Ascaridia galli*	5	2	0.400 ± 0.273	0.00091 ± 0.00054	0.400
	*Toxocara canis*	6	2	0.600 ± 0.129	0.01633 ± 0.00351	7.200
	*Ophidascaris filaria*	3	3	1.000 ± 0.272	0.00452 ± 0.00159	2.000
	*Baylisascaris transfuga*	3	3	1.000 ± 0.272	0.00907 ± 0.00363	4.000
ITS	*Toxascaris leonina*	12	2	0.167 ± 0.134	0.00019 ± 0.00016	0.167
	*Parascaris equorum*	3	2	0.667 ± 0.314	0.00080 ± 0.00038	0.667
	*Ascaridia galli*	6	4	0.800 ± 0.172	0.00339 ± 0.00111	3.333
	*Toxocara canis*	6	6	1.000 ± 0.096	0.03543 ± 0.01040	40.467
	*Ophidascaris filaria*	3	2	0.667 ± 0.314	0.00193 ± 0.00091	2.000
	*Baylisascaris transfuga*	3	2	0.667 ± 0.314	0.00077 ± 0.00036	0.667

*N, number of isolates; S, number of polymorphic sites; H, number of haplotypes; π, Nucleotide diversity; Hd, Haplotype (gene) diversity; K, Average number of nucleotide differences*.

The phylogenetic trees constructed based on the p*cox*1 and ITS genes showed that 32 sample strains of ascarid worms from different host sources (except A2) can cluster with their corresponding species and genera to form a branch. Nematodes in the genus Toxocara are distant relatives of the free-living, soil (and laboratory)-dwelling roundworm Caenorhabditis elegans (24). *T. leonina* of Amur tiger and other felines clustered into one clade, showing a closer relationship than canines, which is identical to the result of Peng et al. (25). *P. equorum* was closely related to *A. suum* and *B. transfuga*, which is a similar result to Gao et al. (26). Interestingly, when building a tree based on the ITS gene, *T. leonina* is closely related to *T. canis, T. cati* and *T. vitulorum*, which is different from building a tree based on the p*cox*1 gene. Scholars have found that it is limited to reconstructing the genetic relationship between species based on a single or a few genes. Due to horizontal gene transfer (HGT), nucleotide substitution saturation, interspecific hybridization and paralog, the inconsistency between a gene tree and a species tree will appear when using a single gene to build trees (27).

## Conclusion

In summary, both p*cox*1 and ITS genes can be used as molecular markers to study the genetic variation of ascarids from wild animals. In addition, the ascarid worm samples were collected from white lions, Amur tigers, South China tigers, cheetahs, zebras, peacocks, wolfs, snakes and bears from Changsha Ecological Zoo in Hunan Province, suggesting that other wild animals raised in this area also have the risk of ascarid infection. Thus, it is necessary to formulate a perfect anthelmintic regime to prevent repeated and recessive infections and reduce the loss caused in wild animals due to ascarid worms.

## Data Availability Statement

The datasets presented in this study can be found in online repositories. The names of the repository/repositories and accession number(s) can be found in the article/Supplementary Material.

## Author Contributions

WL, S-YC, Q-GQ, and T-FG: conceptualization. WL, S-YC, Q-GQ, T-FG, and H-LM: data curation, visualization, and validation. S-YC, T-FG, and H-LM: formal analysis. WL, S-YC, Q-GQ, T-FG, H-LM, J-LH, X-RX, W-CL, and FL: investigation and methodology. S-YC, T-FG, H-LM, and WL: writing original draft preparation. WL and Q-GQ: funding. All authors have read and agreed to the published version of the final manuscript.

## Funding

This project was supported by the Natural Science Foundation of Hunan Province, China (2021JJ30335) and the Horizontal Scientific Research Project of Hunan Province, China (202105000152).

## Conflict of Interest

The authors declare that the research was conducted in the absence of any commercial or financial relationships that could be construed as a potential conflict of interest.

## Publisher's Note

All claims expressed in this article are solely those of the authors and do not necessarily represent those of their affiliated organizations, or those of the publisher, the editors and the reviewers. Any product that may be evaluated in this article, or claim that may be made by its manufacturer, is not guaranteed or endorsed by the publisher.

## Supplementary Material

Supplementary Figure 1 PCR amplification products of mitochondrial p*cox*1 gene of *Ascaris*. M: DL500 DNA Marker; 1-33: Samples of different hosts ascarids; +: Positive; -: Negative.

Supplementary Figure 2 PCR amplification products of mitochondrial ITS gene of *Ascaris*. M: DL500 DNA Marker; 1-33: Samples of different hosts ascarids; +: Positive; -: Negative.

Supplementary Figure 3 Sequence Distance of 33 samples based on p*cox*1 gene.

Supplementary Figure 4 Sequence Distance of 33 samples based on ITS gene.
