# Supplementary figures and images for: Molecular Identification and Phylogenetic Analysis of Ascarids in Wild Animals
Source: Front Vet Sci. 2022 Apr 29;9:891672. doi: 10.3389/fvets.2022.891672 (PMC9100682; doi:10.3389/fvets.2022.891672)

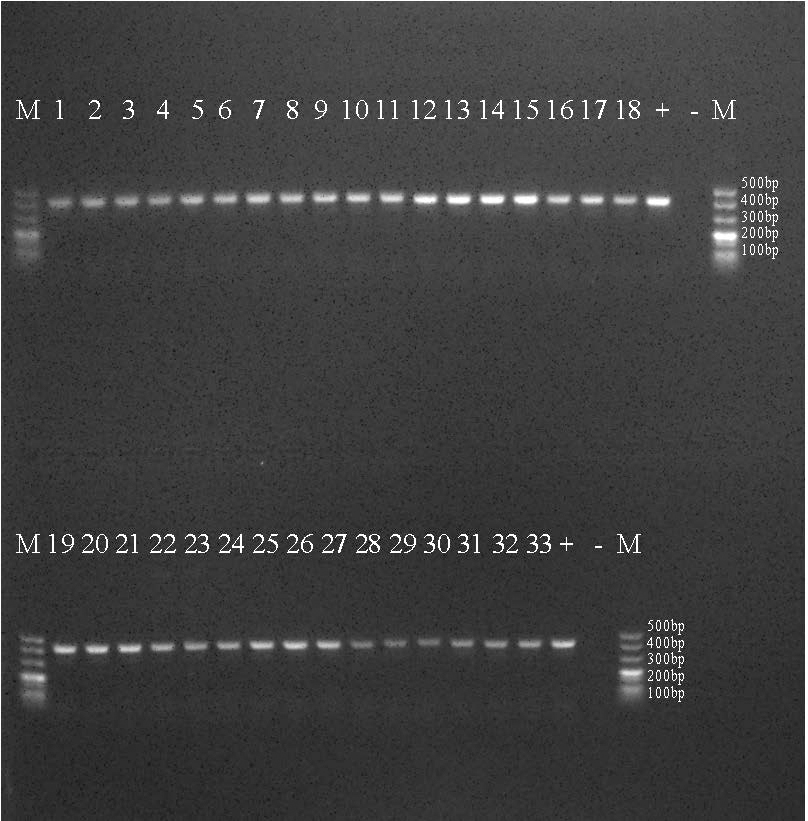

Supplement: Supplementary Figure 1 — PCR amplification products of mitochondrial pcox1 gene of Ascaris. M: DL500 DNA Marker; 1-33: Samples of different hosts ascarids; +: Positive; -: Negative. [file Image_1.JPEG]

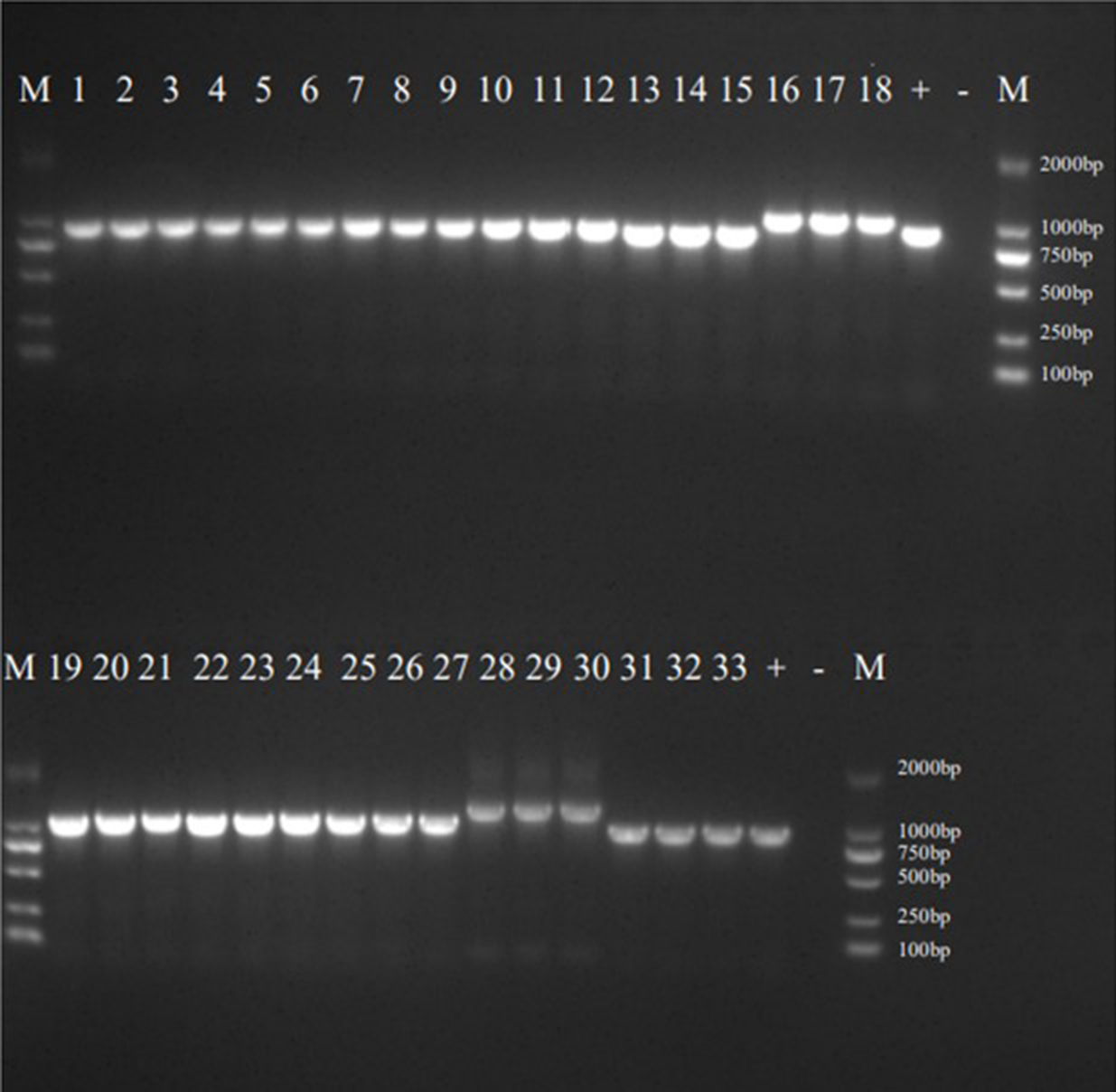

Supplement: Supplementary Figure 2 — PCR amplification products of mitochondrial ITS gene of Ascaris. M: DL500 DNA Marker; 1-33: Samples of different hosts ascarids; +: Positive; -: Negative. [file Image_2.JPEG]

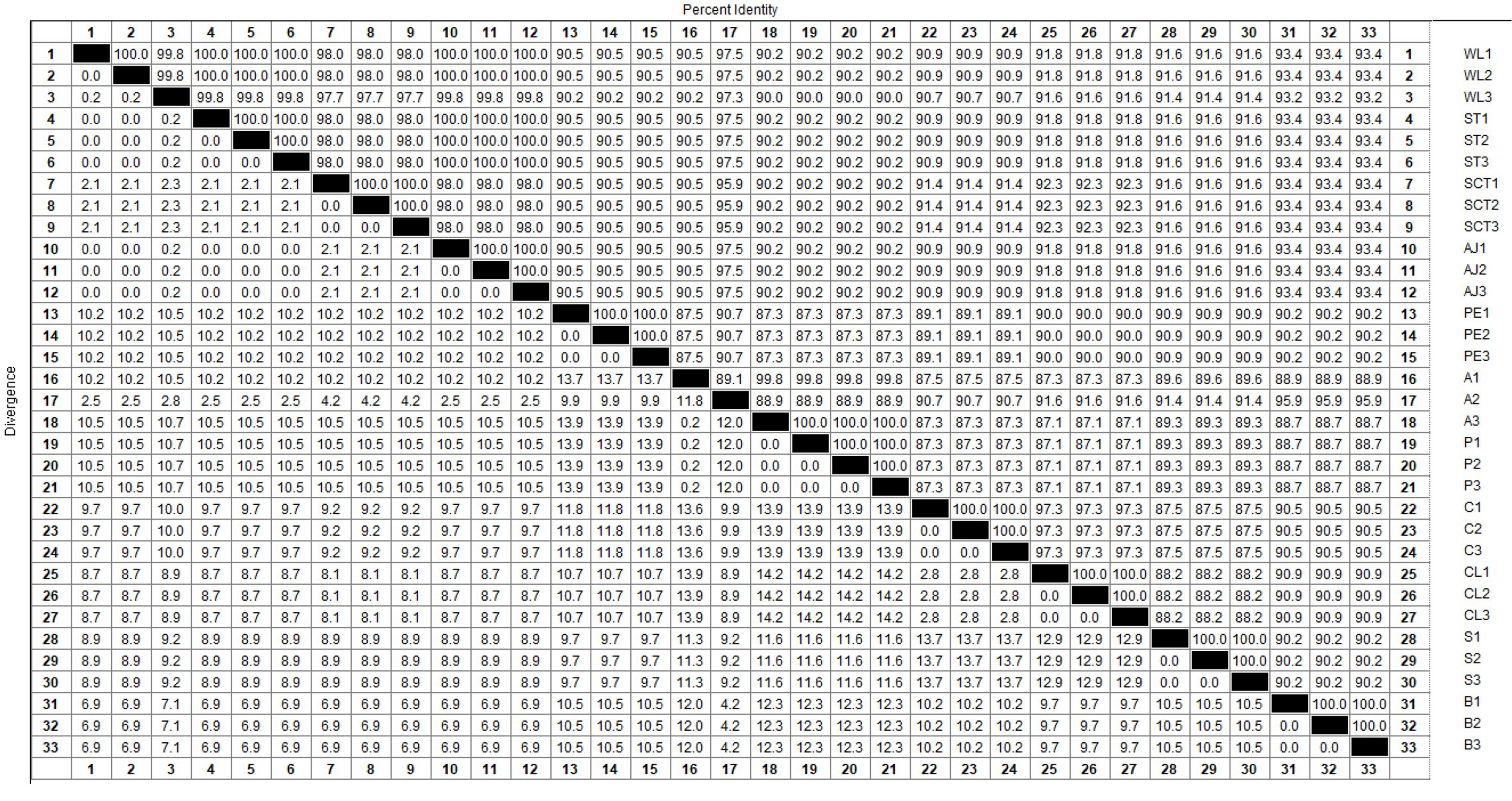

Supplement: Supplementary Figure 3 — Sequence Distance of 33 samples based on pcox1 gene. [file Image_3.JPEG]

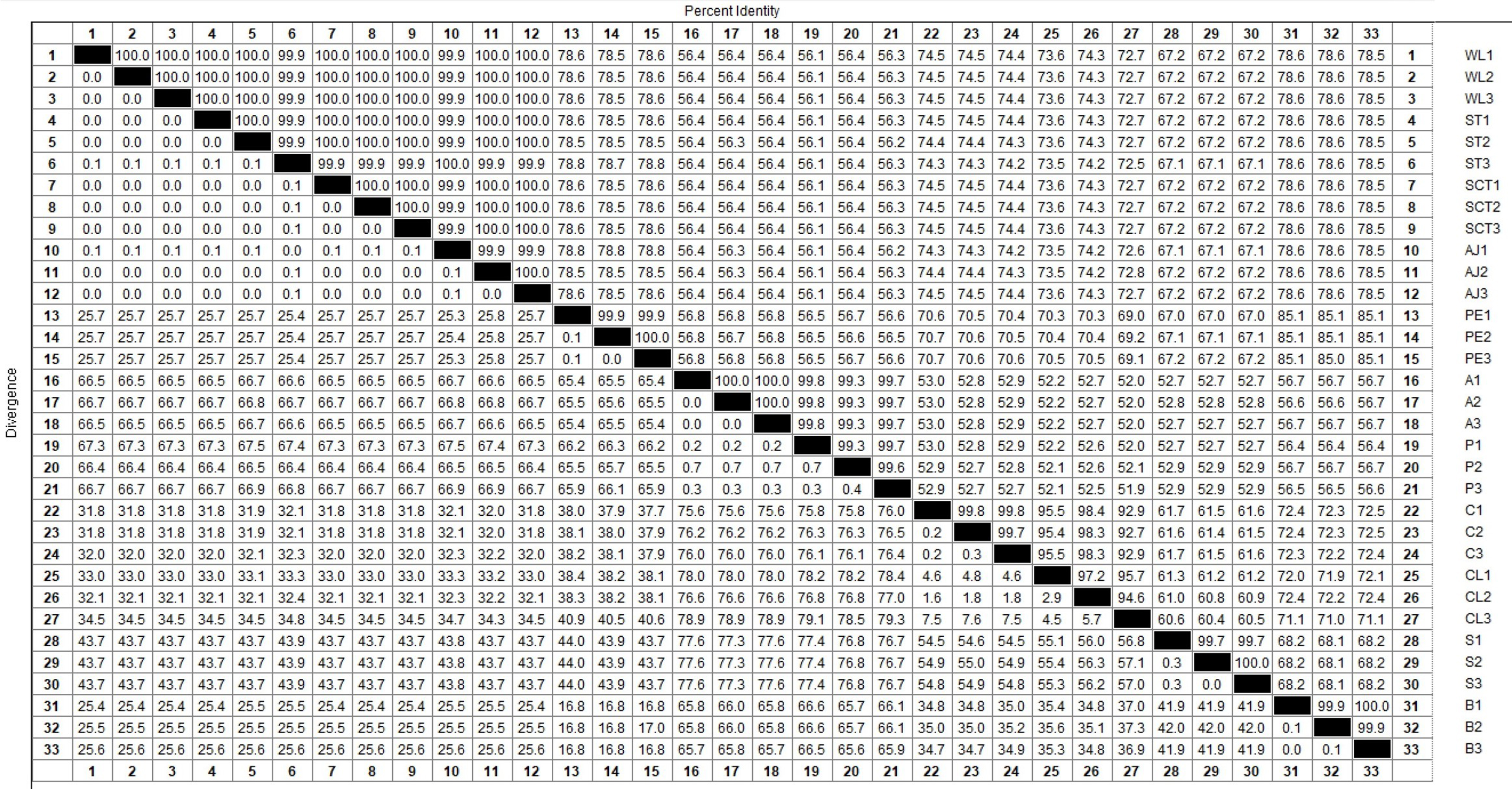

Supplement: Supplementary Figure 4 — Sequence Distance of 33 samples based on ITS gene. [file Image_4.JPEG]

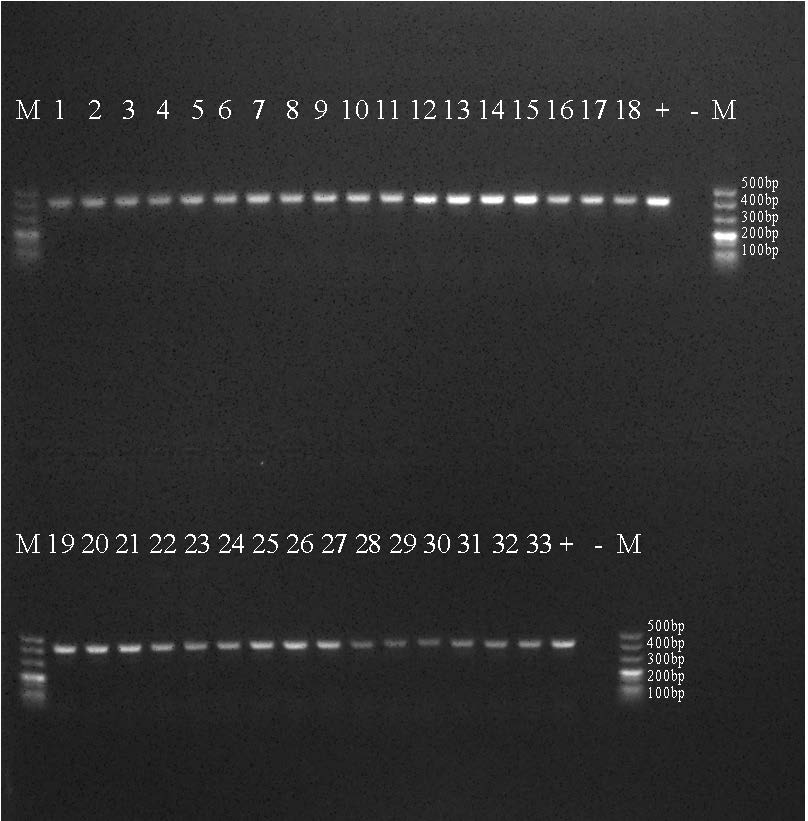

Supplement: Supplementary file 5 [file Data_Sheet_1.ZIP › Raw Data/COX1/Gel electrophoresis results of cox1.jpg]

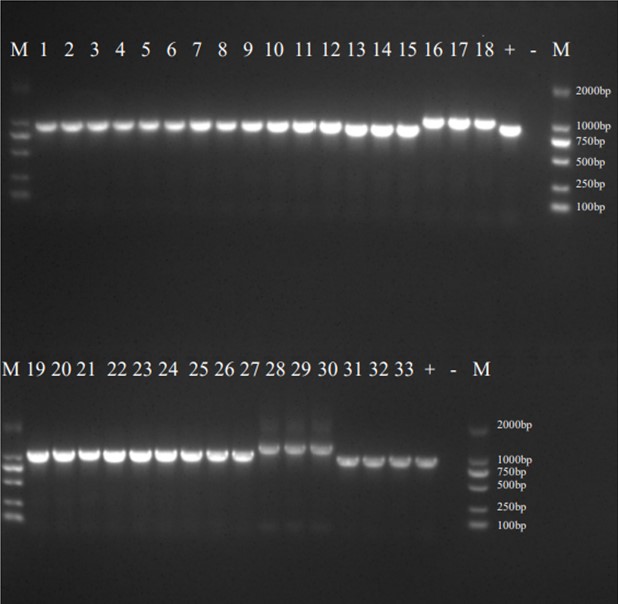

Supplement: Supplementary file 5 [file Data_Sheet_1.ZIP › Raw Data/ITS/Gel electrophoresis results of ITS.jpg]
